# Which TDP-43 aggregates are toxic in ALS?

**DOI:** 10.18632/oncotarget.13461

**Published:** 2016-11-18

**Authors:** Cristiana Valle, Maria Teresa Carrì

**Affiliations:** Department of Biology, University of Rome Tor Vergata, and Fondazione Santa Lucia IRCCS, Rome, Italy

**Keywords:** amyotrophic lateral sclerosis, ALS, TDP-43, protein aggregation, neurodegeneration

Amyotrophic Lateral Sclerosis (ALS) is a fatal, adult onset neurodegenerative disorder with a characteristic phenotype stemming from progressive degeneration of motor neurons and muscle waste. The pathogenesis of ALS is complex and only partially understood, with several possible mechanisms operating in motor neurons and in non-neuronal cells as well. As for other neurodegenerative diseases, mitochondrial damage, oxidative stress, neuroinflammation, excitotoxicity and RNA dis-metabolism contribute to the phenotype, together with mutations in a number of genes that have been proposed as causative or at least as modifier in the pathogenesis of the disease [1]. As in other neurodegenerative diseases, protein aggregates are invariably found in the affected population of neurons in post-mortem samples from patients, regardless their classification as sporadic (sALS) or familial ALS (fALS) cases, although the aggregated protein may vary in different forms of ALS. Protein aggregates may arise as a consequence of improper folding of a mutant protein in fALS, but also following oxidative modifications of crucial proteins, defective chaperons and/or defective Unfolded Protein Response and protein degradation in sALS [2].

On the ground that protein aggregates may represent toxic species directly linked to motor neuron demise, we and others have thoroughly studied the process of formation of inclusions of some ALS-linked proteins. In particular, recent focus has been on studies on the aggregation of TDP-43, a nuclear RNA-binding protein that is found delocalized in cytoplasmic inclusions in all sALS and fALS patients, with the only exception of fALS with SOD1 mutations.

In a recent study we shed new light on the contribution of different species of TDP-43 aggregates in ALS [3]. We demonstrated the existence of two different components, i.e. oligomers and large aggregates, that are made of different molecular species and whose formation is driven by different forces. Large aggregates contain unfolded full length isoforms of the protein, held together mostly by unspecific hydrophobic interactions. However, oligomers formation is a two-step process primed by the oxidation of the two N-terminal cysteine residues and completed through mechanisms involving four other cysteines localized in the RNA-recognition motifs. Interestingly, the two truncated isoforms generated by cleavage of the full length protein and found in neurons of ALS patients [4] are entrapped exclusively in oligomers.

This last evidence opens the question on which TDP-43 isoform is the truly toxic species in the pathogenic process, a question that should be solved before any rationale attempt to use an anti-aggregation approach in patients.

Full length, wild type TDP-43 is found aggregated in the vast majority of sALS and fALS patients; studies *in vivo* and *in vitro* demonstrate that this protein is not particularly prone to aggregation by itself unless it is purified or highly overexpressed (and thus in non-physiological conditions). However, under conditions of chronic, mild oxidative stress mimicking those accompanying ageing, both wild type and mutant TDP-43 have an increased aggregation propensity. Thus, the idea that a loss of function due to entrapment of the aggregated full length protein in the cytosol may take place either as a consequence of mutation or following age-related pro-oxidant conditions is to be considered. This concept is supported by studies demonstrating that the endogenous TDP-43 trapped in the aggregates undergoes ubiquitination and hyperphosphorylation, that are the two most important post-translational modifications seen in pathological TDP-43 inclusions, and that an intact N-terminal domain is necessary for the loss-of-function in the splicing process [5]. If this is the case, then the goal is to intercept the formation of large aggregates in order to prevent the delocalization and loss of function of the protein in patients.

On the other hand, the idea that the short isoforms may represent the toxic species is suggested by the fact that the shorter isoforms are typically found in patients vs. healthy control [4] and by evidence in transgenic mice expressing a C-term shorter isoform [6]. Furthermore, we have observed that when the full length TDP43 is expressed, the shorter proteins form only oligomers that may contribute to the propagation of the disease via a prion-like mechanism as recently suggested [7]. In this case, early interception of the formation of oligomers may constitute a sound approach to prevent not only toxicity of the short isoforms TDP-43 but also spreading of damage to still healthy, neighbor motor neurons.

As a final word of caution, it must be stressed that only overexpression of wild type or mutant protein induces an ALS-like motor phenotype in model animals, and in this respect the toxic effects of endogenous, low level TDP-43 aggregates are still to be conclusively ascertained.

## References

[1] Robberecht W (2013). Nat Rev Neurosci.

[2] Blokhuis AM (2013). Acta Neuropathol.

[3] Bozzo F (2016). Neurobiol Dis.

[4] Neumann M (2006). Science.

[5] Romano V (2015). Prion.

[6] Walker AK (2015). Hum Mol Genet.

[7] Feiler MS (2015). J Cell Biol.

